# Three-Dimensional Analysis of Sex- and Gonadal Status- Dependent Microglial Activation in a Mouse Model of Parkinson’s Disease

**DOI:** 10.3390/ph16020152

**Published:** 2023-01-20

**Authors:** Amandine Isenbrandt, Katherine Coulombe, Marc Morissette, Mélanie Bourque, Jérôme Lamontagne-Proulx, Thérèse Di Paolo, Denis Soulet

**Affiliations:** 1 Centre de Recherche du CHU de Québec, Axe Neurosciences, 2705, Boulevard Laurier, Québec, QC G1V 4G2, Canada; 2 Faculté de Pharmacie, Pavillon Ferdinand-Vandry, Université Laval, 1050, Avenue de la Médecine, Québec, QC G1V 0A6, Canada

**Keywords:** Parkinson’s disease, sex differences, microglial activation, microglial morphology, dutasteride

## Abstract

Parkinson’s disease (PD) is characterized by neurodegeneration and neuroinflammation. PD prevalence and incidence are higher in men than in women and modulation of gonadal hormones could have an impact on the disease course. This was investigated in male and female gonadectomized (GDX) and SHAM operated (SHAM) mice. Dutasteride (DUT), a 5α-reductase inhibitor, was administered to these mice for 10 days to modulate their gonadal sex hormones. On the fifth day of DUT treatment, mice received 1-methyl-4-phenyl-1,2,3,6-tetrahydropyridine (MPTP) to model PD. We have previously shown in these mice the toxic effect of MPTP in SHAM and GDX males and in GDX females on dopamine markers and astrogliosis whereas SHAM females were protected by their female sex hormones. In SHAM males, DUT protected against MPTP toxicity. In the present study, microglial density and the number of doublets, representative of a microglial proliferation, were increased by the MPTP lesion only in male mice and prevented by DUT in SHAM males. A three-dimensional morphological microglial analysis showed that MPTP changed microglial morphology from quiescent to activated only in male mice and was not prevented by DUT. In conclusion, microgliosis can be modulated by sex hormone-dependent and independent factors in a mice model of PD.

## 1. Introduction

Parkinson’s disease (PD) is the second most common neurodegenerative disease [1] and the most prevalent disease with movement disorders [1]. The main neuropathological features of PD are the selective degeneration of dopamine (DA) neurons in the nigrostriatal pathway [2] and the abnormal accumulation and aggregation of alpha-synuclein (α-syn) [3]. The most important risk factor for PD is aging, although this alone cannot explain the appearance of this disease [4]. Exposure to certain pollutants, particularly herbicides and pesticides, represents a significant risk factor and is associated with epidemiological observations showing a higher prevalence of PD among farmers and people living in rural areas [5]. Sex is also a risk factor for PD. Indeed, epidemiological studies report a higher prevalence and earlier age of onset in men [6,7,8]. Additional risk factors include nutrient intake, alcohol consumption and body mass index as well as other diseases, such as diabetes or cancer, and having already suffered from one or more traumatic brain injuries [9]. Finally, genetic mutations also represent a risk factor for the familial form of PD and for the sporadic form, considering that it could increase sensitivity to environmental factors as mentioned above [9].

PD is characterized by motor symptoms that emerge after the loss of more than 80% of DA neurons in the substantia nigra pars compacta (SNpc) [10]. The clinical evaluation of these symptoms allows the diagnosis of PD [10].

There is no curative treatment for PD. Existing treatments are symptomatic and do not influence the cause or the progression of PD. The most widely used treatment is levodopa (L-Dopa), the immediate precursor of DA to replace DA deficiency caused by this disease [11]. However, after years of treatment, L-Dopa causes serious side effects, such as dyskinesia, and loses its efficacy [12]. It is therefore of interest to study potential therapeutic action of new or existing drugs to prevent, delay and/or stop PD.

During PD, microglia become highly activated [13]. Phagocytose is the microglia’s main function in the brain—to eliminate cells or cellular debris [14]. In the early stage of PD, microglia may not be detrimental and participate in the scavenging of abnormal deposits of α-syn as well as in neuronal homeostasis [15]. As the disease progresses with the death of DA neurons in the SNpc, a strong microglial activation was found in PD patient’s post-mortem tissues [16]. In a PD mouse model using the 1-methyl-4-phenyl-1,2,3,6 tetrahydropyridine (MPTP) toxin, microglial activation has also been confirmed by important morphological changes in the SNpc [17,18]. Indeed, microglial activation is characterized by a change from a quiescent highly ramified morphology into an activated ameboid shape [19].

Once activated, a microglia produces a large range of inflammatory mediators, such as proinflammatory cytokines (e.g., TNF-α, IL-6, IL-1β, IFN-γ…), chemokines and reactive oxygen species [15,20,21,22,23,24]. Excessive accumulation of these mediators will lead to the death of DA neurons in the SNpc as reported by positron emission tomographic (PET) analysis in living PD patients [25,26]. Microglia can be activated by aggregated α-syn secreted from neurons [27], triggering a pro-inflammatory response, thus accelerating PD progression [14,28]. Moreover, microglia might directly participate in neuronal cell death during PD by the aberrant engulfing of neurons [15].

Epidemiological observations report that the incidence and prevalence of PD are 1.5 to 2-fold higher in men than in women [29,30,31,32,33,34,35,36,37,38] and that the disease begins about 2.2 years later in women [39]. Women also present tremors more often, which are associated with the deterioration of motor skills and striatal degeneration as well as the slower progression of PD [40]. Studies show the onset of PD symptoms may be delayed by higher physiological levels of striatal DA in women, seemingly via the neuroprotective activity of estrogen [39]. These initially higher DA levels could delay reaching the critical threshold for DA depletion and therefore delay the development of PD symptoms. The loss of DA required for the onset of symptoms in men and women would, therefore, be relatively similar [39]. However, women do not seem to have advantages over men once diagnosed. Indeed, once the critical threshold for striatal DA depletion is exceeded, the progression of PD does not differ between men and women [39]. This suggests that estrogen may exert some neuroprotection during the preclinical stage of PD, with a time difference in the onset of the degenerative process, but that neuroprotection will no longer be possible once symptoms become clinically apparent [39]. The susceptibilities to the various risk factors also vary between sexes. Indeed, the risk factors that are the most important in women would mainly be physical inactivity while for men it would be chronic stress at work, cholesterol and advanced age [41,42]. With this data, it does not seem surprising that the male gender represents a risk factor for PD [40]. Sex differences also exist in the susceptibility to developing certain symptoms of PD. As mentioned previously, women would show more tremors, but also more postural instability [40,43], than men, and the latter would show more rigidity and bradykinesia [44]. For non-motor symptoms of PD, differences are also reported. Women more often report stress, depression and gastrointestinal disturbances while men complain more of excessive salivation and hyposmia [45]. In women, during the week before menstruation, when estrogen levels drop 50–60%, women report worsening of symptoms [46], further suggesting the protective effect of estrogen. As a result, studies were carried out observing factors that increase exposure to estrogens during life (number of pregnancies, early age of menarche, advanced age of menopause, use of exogenous estrogens) and an association was found between these events and protection against the development of PD [47,48,49,50].

Recently, a study with a Mendelian randomization approach showed that the later onset of menopause was associated with lowering PD risk in women, highlighting again the potential neuroprotective role of sex hormones and other factors related to the menopausal state [51]. Sex differences have already been reported for 6-hydroxydopamine (6-OHDA) [52,53] and MPTP [53,54,55] animal models of PD with protection in females.

Microglia was shown to participate in the masculinization of dendritic spine density induced by estradiol. Microglia was also reported to be essential in the feedforward process during which prostaglandin production is upregulated by estradiol during the hormone-induced process of brain masculinization occurring with the surge of male gonadal activity in neonatal phase [56]. Testosterone produced by the testis is aromatized to estradiol, the main masculinizing hormone in rodent brains [14,57,58]. This masculinization allows the brain architecture organization [59,60] and leads to characteristic neuroanatomical sexual differences in the rodent brain [14,57,58]. After birth, treating females with estradiol will lead to a masculine microglial phenotype [14,57,58].

While sex hormones seem to be able to act on the microglial phenotype, biological sex also seems to determine this phenotype. In a study investigating sex-specific features in microglia from adult mice, microglia isolated from the adult brain were transplanted in the brain of other mice [61].When male microglia were transplanted in male mice, permanent middle cerebral artery occlusion (pMCAO) led to a large brain lesion in these mice. However, when male mice were transplanted with female microglia, this led to a smaller lesion showing the conservation of sex-specific features knowing that female microglia are protective because of their potential to restrict damages caused by this type of intervention [61].

With these sex differences and the known participation of female sex hormones in neuroprotection in PD, it seems relevant to test drugs capable of raising levels of female sex hormones for their possible protective effect in PD. Therefore, we investigated Dutasteride’s (DUT) therapeutic potential. DUT is a 5α-reductase enzyme inhibitor used to treat male androgenic alopecia [62] and to relieve symptoms of benign prostatic hyperplasia [63]. This enzyme is responsible for converting testosterone into dihydrotestosterone and progesterone into 5α-dihydroprogesterone [64]. Interestingly, inhibiting the 5α-reductase enzyme will lead to progesterone and testosterone accumulation. Testosterone could then be converted into estrogen by the enzyme aromatase. This leads to an increase in female sex hormones known to be neuroprotective [7,8]. We previously reported that the treatment of male mice with DUT exerts protective properties against MPTP toxicity [65].

In a previous paper [55], we used male and female, gonadectomized (GDX mice) or sham operated (SHAM mice) mice to investigate the sex differences and the impact of gonadal status on MPTP-induces mouse model of PD. GDX mice were used to model the andropause and menopause status as well as to investigate the role of gonadal sex hormones and their withdrawal. MPTP decreased DA and DA metabolites contents, DA transporter and vesicular monoamine transporter 2 as well as astrocyte activation in all groups except in SHAM females, and in SHAM males, DUT was protective [55]. We showed a protective effect of female gonadal sex hormones in this PD model and protection by DUT in SHAM males [55].

The present study examined the implication of microglia in the neuroprotective effect of female gonadal hormones and DUT on DA parameters and compared them to astrocytic activation [55]. The aim of this study was to assess microglial activation with measure of striatal microglial density and morphology using a three-dimensional analysis.

## 2. Results

### 2.1. MPTP Caused a Decrease in Striatal Tyrosine Hydroxylase (TH) Intensity in All Group except in SHAM Females and DUT Prevented this Toxic Effect Only in SHAM Male Mice

**Figure 1:**
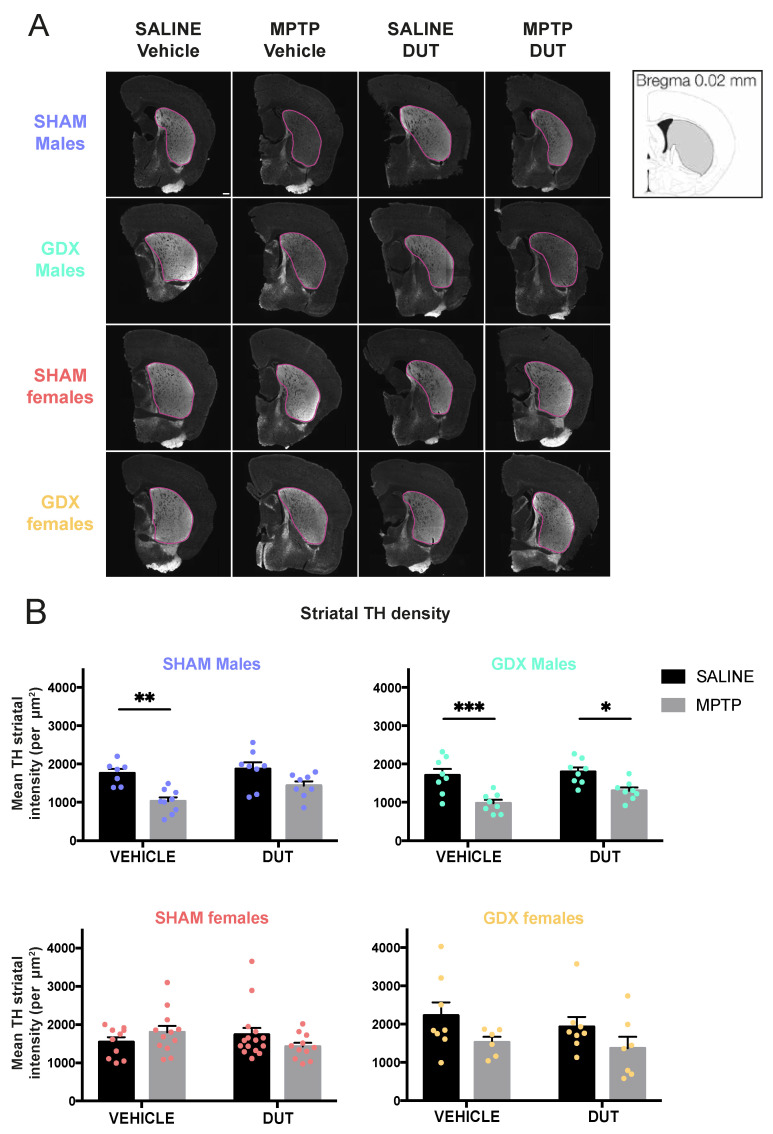
Effect of mice gonadal status (SHAM, GDX), MPTP, and dutasteride (DUT) treatment on TH striatal levels. (**A**) Representative example of striatal TH immunofluorescence. Scale bar = 1000 μm and (**B**) TH striatal intensity in SHAM and GDX males and SHAM and GDX female mice. Values shown are the means pixel intensity ± SEM of 6-16 mice per group. Two-way ANOVA, Tukey post-hoc test: * *p* < 0.05, ** *p* < 0.01 and *** *p* < 0.001.

TH^+^ intensity was measured in the striatum to assess dopaminergic projections as an indicator of neurodegeneration. Examples of TH immunofluorescence in the striatum of the experimental groups are shown in Figure 1A and their mean TH^+^ intensity in Figure 1B. Under basal conditions, striatal TH immunofluorescence levels were not different between male and female SHAM and GDX mice (Sex: F (1, 29) = 0.5253, *p* = 0.4744; Gonadal status: F (1, 29) = 2.239, *p* = 0.1453; interaction F (1, 29) = 3.129, *p* = 0.0874).

In SHAM male mice (*n* = 32), striatal TH^+^ intensity was significantly changed (MPTP: F (1, 28) = 20.68, *p* < 0.0001; DUT: F (1, 28) = 4.05, *p* = 0.0538; interaction: F (1, 28) = 1.33, *p* = 0.2581). MPTP caused a 42% loss of striatal TH^+^ intensity in vehicle-treated SHAM male mice (*p* = 0.0022 vs. vehicle saline) whereas dutasteride-treated MPTP SHAM male mice had no significant loss (*p* = 0.0985 vs. DUT saline). DUT left striatal TH^+^ intensity unchanged in saline treated SHAM male mice.

GDX male mice (*n* = 33) also showed significant changes of TH^+^ intensity (MPTP: F (1, 28) = 26.33, *p* < 0.0001; DUT: F (1, 28) = 3.064, *p* = 0.0910; interaction: F (1, 28) = 0.9682, *p* = 0.3336). MPTP caused a 43% loss of striatal TH^+^ intensity in vehicle-treated GDX male mice (*p* = 0.0010 vs. vehicle saline) and 27% in dutasteride-treated GDX male mice (*p* = 0.0317 vs. DUT saline).

SHAM females (*n* = 48) showed no significant changes of TH^+^ intensity (MPTP: F (1, 44) = 0.03709, *p* = 0.8482; DUT: F (1, 44) = 0.3309, *p* = 0.5681; interaction: F (1, 44) = 3.339, *p* = 0.0744) as well as in GDX females (*n* = 28) (MPTP: F (1, 24) = 3.613, *p* = 0.0694; DUT: F (1, 24) = 0.9435, *p* = 0.3411; interaction: F (1, 24) = 0.1033, *p* = 0.7507). Nevertheless, MPTP caused a non-significant 31% reduction of TH^+^ intensity in vehicle-treated GDX female mice (*p* = 0.7006 vs. vehicle saline) and 29% in DUT-treated GDX female mice (*p* = 0.7004 vs. DUT saline).

### 2.2. MPTP Caused an Increase in Striatal Microglial Density in All Groups Except in SHAM Females, and DUT Prevented this Effect Only in SHAM Male Mice

**Figure 2:**
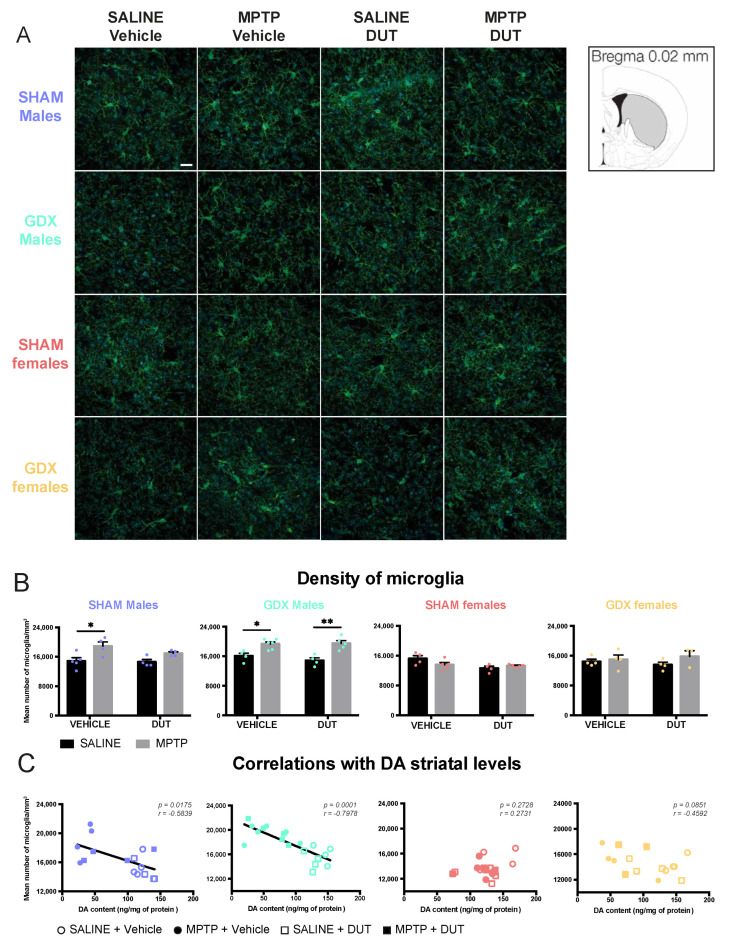
Effect of mice gonadal status (SHAM, GDX), MPTP, and dutasteride (DUT) treatment on striatal microglial density. (**A**) Representative example of striatal Iba1 immunofluorescence. Scale bar = 20 μm; (**B**) Iba1 positive cells count in the striatum in SHAM and GDX males and SHAM and GDX female mice. Values shown are the mean number of Iba1 positive cells per mm^3^ ± SEM of 4 images of 4-6 mice per group and 7 to 25 microglia per image. Two-way ANOVA, Tukey post-hoc test: * *p* < 0.05 and ** *p* < 0.01 and (**C**) Correlations between striatal DA contents with striatal microglial density (Iba1 positive cell counts).

An Iba1^+^ cell count was measured to assess microglial activation and as an indicator of neuroinflammation. Examples of Iba1 immunofluorescence in the striatum of the experimental groups are shown in Figure 2A, Figure S1, Video S1 and their mean Iba1 + cell count in Figure 2B. Under basal conditions, striatal Iba1^+^ cell counts were not different between male and female SHAM and GDX mice (Sex: F (1, 13) = 0.636, *p* = 0.4395; Gonadal status: F (1, 13) = 0.07415, *p* = 0.7897; interaction F (1, 13) = 1.55, *p* = 0.2352).

In SHAM male mice (*n* = 17), striatal Iba1^+^ cell counts were significantly changed (MPTP: F (1, 13) = 13.7, *p* = 0.0027; DUT: F (1, 13) = 1.659, *p* = 0.2201; interaction F (1, 13) = 0.9599, *p* = 0.3451). MPTP caused a 27% increase of striatal Iba1^+^ cell counts in vehicle-treated SHAM male mice (*p* = 0.0216 vs. vehicle saline) whereas dutasteride-treated SHAM male mice had no significant increase (*p* = 0.2847 vs. DUT saline). DUT left unchanged striatal Iba1^+^ cell counts in saline treated SHAM male mice.

GDX male mice (*n* = 19) also showed significant changes of Iba1^+^ cell counts (MPTP: F (1, 15) = 31.85, *p* < 0.0001; DUT: F (1, 15) = 0.5892, *p* = 0.4547; interaction: (1, 15) = 1.044, *p* = 0.3231). MPTP caused a 20% increase in striatal Iba1^+^ cell counts in vehicle-treated GDX male mice (*p* = 0.0211 vs. vehicle saline) and 31% in dutasteride-treated GDX male mice (*p* = 0.0017 vs. DUT saline).

SHAM females (*n* = 18) showed no significant changes in Iba1^+^ cell counts (MPTP: F (1, 14) = 0.8292, *p* = 0.3779; DUT: F (1, 14) = 6.43, *p* = 0.0238; interaction (1, 14) = 4.357, *p* = 0.556) as well as in GDX females (*n* = 15) (MPTP: F (1, 11) = 1.909, *p* = 0.1945; DUT: F (1, 11) = 0.0006547, *p* = 0.9800; interaction: F (1, 11) = 0.7129, *p* = 0.4165).

Figure 2C shows correlations between striatal DA contents previously reported for these mice [55] and their number of microglial cell counts in the striatum. A significant negative correlation was observed between individual mouse values of DA striatal contents and Iba1 positive cell count of SHAM male mice and GDX male mice but not in the SHAM and GDX female mice.

### 2.3. MPTP Caused an Increase in Striatal Microglial Doublet Count in All Experimental Groups except in SHAM Females, and DUT Prevented This Effect Only in SHAM Male Mice

**Figure 3:**
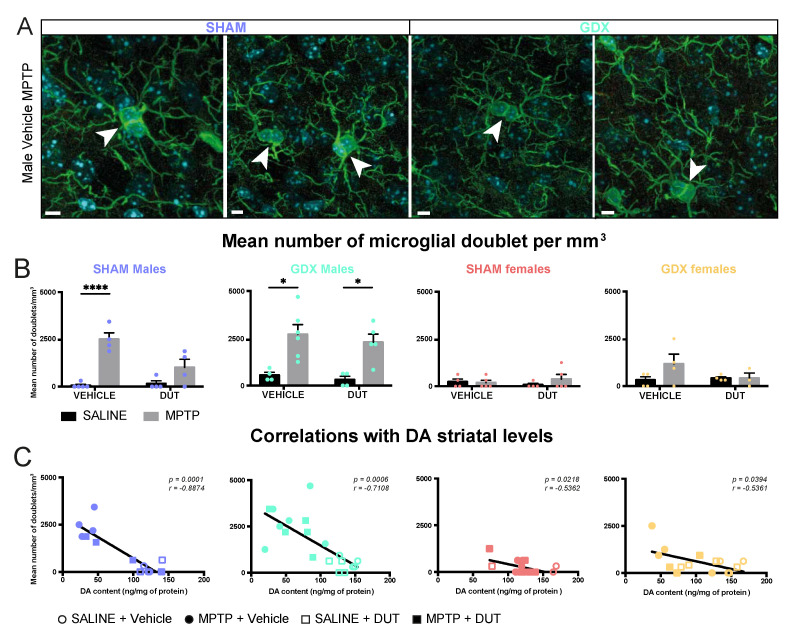
Effect of mice gonadal status (SHAM, GDX), MPTP, and dutasteride (DUT) treatment on striatal microglial doublet. (**A**) Representative example of striatal microglial doublet indicated with white arrowheads. Scale bars = 5 μm; (**B**) Microglial doublet counts in the striatum in SHAM and GDX males and SHAM and GDX female mice. Values shown are the mean number of doublets per image ± SEM 4 images of 4-6 mice per group and from 0 to 4 microglial doublet per image. Two-way ANOVA, Tukey post-hoc test: * *p* < 0.05 and **** *p* < 0.0001 and (**C**) Correlations between striatal DA contents with striatal microglial doublet counts.

Iba1 positive cells with a doublet were counted to assess microglial division and as an indicator of neuroinflammation. Examples of microglial doublets with Iba1 immunofluorescence in the striatum of the experimental groups are shown in Figure 3A, Video S2 and their mean Iba1^+^ doublet cell counts in Figure 3B. Sex and gonadectomy affected the density of striatal microglia doublets (Sex: F (1, 15) = 9.862, *p* = 0.0067; Gonadal status: F (1, 15) = 14.8, *p* = 0.0016; interaction F (1, 15) = 13.15, *p* = 0.0025). The striatal microglial doublet count showed no statistical difference between SHAM male and female mice. GDX in female mice left unchanged striatal microglia doublet density whereas male mice had a higher density than SHAM male mice (*p* = 0.0002), SHAM female (*p* = 0.0008) and GDX female (*p* = 0.0010).

In SHAM male mice (*n* = 17), striatal Iba1^+^ doublet cell counts were significantly changed (MPTP: F (1, 13) = 37.56, *p* < 0.0001; DUT: F (1, 13) = 6.683, *p* = 0.0226; interaction F (1, 13) = 8.606, *p* = 0.0116). MPTP caused a 300% increase in striatal Iba1^+^ doublet cell counts in vehicle-treated SHAM male mice (*p* < 0.0001 vs. vehicle saline) whereas dutasteride-treated SHAM male mice had no significant increase (*p* = 0.1739 vs. DUT saline). DUT left striatal Iba1^+^ doublet cell counts unchanged in saline treated SHAM male mice.

GDX male mice (*n* = 19) also showed significant changes in Iba1^+^ doublet cell counts (MPTP: F (1, 15) = 25.06, *p* = 0.0002; DUT: F (1, 15) = 0.5947, *p* = 0.4526; interaction: F (1, 15) = 0.04663, *p* = 0.8319). MPTP caused a 395% increase in striatal Iba1^+^ doublet cell counts in vehicle-treated GDX male mice (*p* = 0.0104 vs. vehicle saline) and 633% in dutasteride-treated GDX male mice (*p* = 0.0246 vs. DUT saline).

SHAM females (*n* = 18) showed no significant changes in Iba1^+^ doublet cell counts (MPTP: F (1, 14) = 0.5105, *p* = 0.4866; DUT: F (1, 14) = 0.007977, *p* = 0.9301; interaction: F (1, 14) = 0.9652, *p* = 0.3425) as well as in GDX females (*n* = 15) (MPTP: F (1, 11) = 1.825, *p* = 0.2039; DUT: F (1, 11) = 1.047, *p* = 0.3281; interaction: F (1, 11) = 1.825, *p* = 0.2039).

Figure 3C shows correlations between striatal DA contents of these mice previously reported [55] and their number of microglial doublets in the striatum. A significant negative correlation was observed between individual mouse values of DA striatal contents and Iba1 positive doublet cell counts in all four gonadal conditions, male and female SHAM and GDX mice.

### 2.4. MPTP Lead to an Activated Phenotype of Striatal Microglia in Male Mice Only

**Figure 4:**
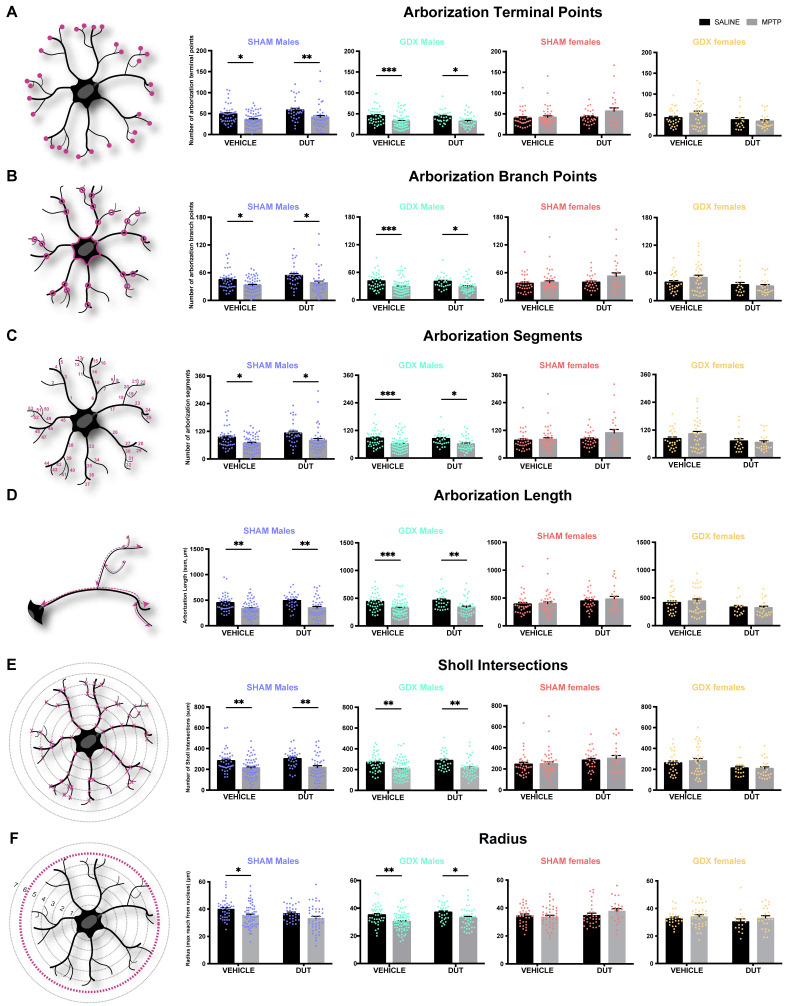
Three-dimension morphological analysis of striatal microglia schematic and effect of mice gonadal status (SHAM, GDX), MPTP, and dutasteride (DUT) treatment on striatal microglial morphology in 3D. (**A**) Number of arborization terminal points representing the number of ramifications terminal points in the whole microglia. Values shown are the sum of terminal points of microglia ± SEM (**B**) Number of arborization branch points representing the number of ramifications branching points in the whole microglia. Values shown are the sum of branch points of microglia ± SEM; (**C**) Number of arborization segments representing the number of ramification segments in the whole microglia. Values shown are the sum of the segment of microglia ± SEM; (**D**) Arborization length defined as the sum of the lengths of all branches within the entire microglia. Values shown are the sum of the length of each branch of each microglia ± SEM; (**E**) Sholl intersections defined as the sum of branches intersections with concentric circles (spheres in 3D) spaced 1 μm each. Values shown are the sum of Sholl intersection of each microglia ± SEM; (**F**) Radius expressed as maximum reach from the nucleus where the concentric circle (spheres in 3D) presenting the intersection of the branches. Values shown are concentric circle numbers having the last branch intersection of microglia. The mean and SEM of 4-6 mice per group and from 1 to 14 microglia per mice is shown. Each point represents one microglia. Two-way ANOVA, Tukey post-hoc test: * *p* < 0.05, ** *p* < 0.01 and *** *p* < 0.001.

A microglial three-dimension morphological analysis was conducted to assess microglial activation status, as shown in Figure 4. Activated microglia have a singular morphology with ameboid nucleus and shortened arborization while quiescent microglia have a small nucleus with long and more ramified arborization. Microglia was chosen as the biological unit for this measurement, and the results are presented as the means for all microglia from three to six mice per group. One 8.01 × 105 μm^3^ slice per mice containing 1 to 15 microglia were reconstructed. The results are shown per mm^3^.

#### 2.4.1. MPTP Caused a Decrease in the Striatal Number of Microglia Arborization Terminal Points in Male Mice

Figure 4A shows the number of arborization terminal points of each microglia. Under basal conditions, the striatal number of arborization terminal points was not different between male and female SHAM and GDX mice (Sex: F (1, 137) = 2.699, *p* = 0.1027; Gonadal status: F (1, 137) = 0.009802, *p* = 0.9213; interaction F (1, 137) = 0.9435, *p* = 0.3331).

In SHAM male mice (*n* = 17), the number of terminal points was significantly changed (MPTP: F (1, 173) = 18.13, *p* < 0.0001; DUT: F (1, 173) = 4.456, *p* = 0.0362; interaction F (1, 173) = 0.3049, *p* = 0.5815). MPTP caused a 27% increase in the number of terminal points in vehicle-treated SHAM male mice (*p* = 0.0259 vs. vehicle saline) and a 29% increase in dutasteride-treated SHAM male mice (*p* = 0.0095 vs. DUT saline). DUT left the microglial number of terminal points unchanged in saline treated SHAM male mice. GDX male mice (*n* = 19) also showed significant changes in the number of terminal points (MPTP: F (1, 181) = 24.68, *p* < 0.0001; DUT: F (1, 181) = 0.09637, *p* = 0.7566; interaction: (1, 181) = 0.05917, *p* = 0.8081). MPTP caused a 29% increase in the number of terminal points in vehicle-treated GDX male mice (*p* = 0.0003 vs. vehicle saline) and 27% in dutasteride-treated GDX male mice (*p* = 0.0141 vs. DUT saline). SHAM females (*n* = 18) showed no significant change in the number of terminal points (MPTP: F (1, 122) = 3.246, *p* = 0.0741; DUT: F (1, 122) = 3.675, *p* = 0.0576; interaction (1, 122) = 1.834, *p* = 0.1781) as well as in GDX females (*n* = 15) (MPTP: F (1, 101) = 0.5601, *p* = 0.4569; DUT: F (1, 101) = 5.302, *p* = 0.0233; interaction: F (1, 101) = 1.843, *p* = 0.1776).

#### 2.4.2. MPTP Caused a Decrease in the Striatal Number of Microglia Arborization Branch Points in Male Mice

Figure 4B shows the number of arborization branch points of each microglia. Under basal conditions, the striatal number of arborization branch points were not different between male and female SHAM and GDX mice (Sex: F (1, 137) = 1.987, *p* = 0.1609; Gonadal status: F (1, 137) = 0.01076, *p* = 0.9175; interaction F (1, 137) = 0.9592, *p* = 0.3291).

In SHAM male mice (*n* = 17), the number of branch points was significantly changed (MPTP: F (1, 173) = 16.83, *p* < 0.0001; DUT: F (1, 173) = 4.161, *p* = 0.0429; interaction F (1, 173) = 0.3586, *p* = 0.5501). MPTP caused a 27% increase in the number of branch points in vehicle-treated SHAM male mice (*p* = 0.0396 vs. vehicle saline) and a 30% increase in dutasteride-treated SHAM male mice (*p* = 0.0119 vs. DUT saline). DUT left unchanged the microglial number of branch points in saline treated SHAM male mice. GDX male mice (*n* = 19) also showed significant changes in the number of branch points (MPTP: F (1, 181) = 23.67, *p* < 0.0001; DUT: F (1, 181) = 0.04304, *p* = 0.8359; interaction: (1, 181) = 0.02404, *p* = 0.8770). MPTP caused a 29% increase in the number of branch points in vehicle-treated GDX male mice (*p* = 0.0006 vs. vehicle saline) and 28% in dutasteride-treated GDX male mice (*p* = 0.0146 vs. DUT saline). SHAM females (*n* = 18) showed no significant change of number of branch points (MPTP: F (1, 122) = 2.922, *p* = 0.0899; DUT: F (1, 122) = 3.26, *p* = 0.0735; interaction (1, 122) = 1.623, *p* = 0.2051) as well as in GDX females (*n* = 15) (MPTP: F (1, 101) = 0.5651, *p* = 0.4539; DUT: F (1, 101) = 5.801, *p* = 0.0178; interaction: F (1, 101) = 1.766, *p* = 0.1869).

#### 2.4.3. MPTP Caused a Decrease in the Striatal Number of Microglia Arborization Segment in Male Mice

Figure 4C shows the number of arborization segments of each microglia. Under basal conditions, striatal number of arborization segments were not different between male and female SHAM and GDX mice (Sex: F (1, 137) = 2.338, *p* = 0.1286; Gonadal status: F (1, 137) = 0.01028, *p* = 0.9194; interaction F (1, 137) = 0.9523, *p* = 0.3308).

In SHAM male mice (*n* = 17), the number of segments was significantly changed (MPTP: F (1, 172) = 16.57, *p* < 0.0001; DUT: F (1, 172) = 4.858, *p* = 0.0288; interaction F (1, 172) = 0.2062, *p* = 0.6503). MPTP caused a 27% increase in the number of segments in vehicle-treated SHAM male mice (*p* = 0.0307 vs. vehicle saline) and a 28% increase in dutasteride-treated SHAM male mice (*p* = 0.0172 vs. DUT saline). DUT left the microglial number of segments unchanged in saline treated SHAM male mice. GDX male mice (*n* = 19) also showed a significant change in the number of segments (MPTP: F (1, 181) = 24.22, *p* < 0.0001; DUT: F (1, 181) = 0.06765, *p* = 0.7951; interaction: (1, 181) = 0.04004, *p* = 0.8416). MPTP caused a 29% increase in the number of segments in vehicle-treated GDX male mice (*p* = 0.0004 vs. vehicle saline) and 27% in dutasteride-treated GDX male mice (*p* = 0.0142 vs. DUT saline).

SHAM females (*n* = 18) showed no significant change in the number of segments (MPTP: F (1, 122) = 3.087, *p* = 0.0814; DUT: F (1, 122) = 3.471, *p* = 0.0649; interaction (1, 122) = 1.731, *p* = 0.1908) as well as in GDX females (*n* = 15) (MPTP: F (1, 101) = 0.563, *p* = 0.4538; DUT: F (1, 101) = 5.548, *p* = 0.0204; interaction: F (1, 101) = 1.806, *p* = 0.1820).

#### 2.4.4. MPTP Caused a Decrease in the Striatal Microglia Arborization Length in Male Mice

Figure 4D shows the total arborization length of each microglia. Under basal conditions, the striatal number of arborization length was not different between male and female SHAM and GDX mice (Sex: F (1, 137) = 2.08, *p* = 0.1515; Gonadal status: F (1, 137) = 0.05284, *p* = 0.8185; interaction F (1, 137) = 0.3921, *p* = 0.5323).

In SHAM male mice (*n* = 17), the length of arborization was significantly changed (MPTP: F (1, 173) = 25.28, *p* < 0.0001; DUT: F (1, 173) = 1.2, *p* = 0.2748; interaction F (1, 173) = 0.3265, *p* = 0.5685). MPTP caused a 25% increase in the arborization length in vehicle-treated SHAM male mice (*p* = 0.0048 vs. vehicle saline) and a 29% increase in dutasteride-treated SHAM male mice (*p* = 0.0018 vs. DUT saline). DUT left the microglial arborization length unchanged in saline treated SHAM male mice. GDX male mice (*n* = 19) also showed significant changes in the arborization length (MPTP: F (1, 181) = 28.21, *p* < 0.0001; DUT: F (1, 181) = 0.8228, *p* = 0.3656; interaction: (1, 181) = 0.03604, *p* = 0.8496). MPTP caused a 27% increase in arborization length in vehicle-treated GDX male mice (*p* = 0.0004 vs. vehicle saline) and 27% in dutasteride-treated GDX male mice (*p* = 0.0028 vs. DUT saline). SHAM females (*n* = 18) showed no significant change in the arborization length (MPTP: F (1, 122) = 0.4821, *p* = 0.4888; DUT: F (1, 122) = 3.482, *p* = 0.0644; interaction (1, 122) = 0.1212, *p* = 0.7284) as well as in GDX females (*n* = 15) (MPTP: F (1, 101) = 0.06592, *p* = 0.7979; DUT: F (1, 101) = 7.418, *p* = 0.0076; interaction: F (1, 101) = 0.2723, *p* = 0.6030).

#### 2.4.5. MPTP Caused a Decrease in the Number of Striatal Microglia Sholl Intersection Points in Male Mice

Figure 4E shows the number of Sholl intersections of each microglia. Under basal conditions, striatal number of Sholl intersections were not different between male and female SHAM and GDX mice (Sex: F (1, 137) = 1.688, *p* = 0.1961; Gonadal status: F (1, 137) = 0.005701, *p* = 0.9399; interaction F (1, 137) = 0.7323, *p* = 0.3936).

In SHAM male mice (*n* = 17), the number of Sholl intersections was significantly changed (MPTP: F (1, 173) = 24.26, *p* < 0.0001; DUT: F (1, 173) = 0.6923, *p* = 0.4065; interaction F (1, 173) = 0.2261, *p* = 0.6350). MPTP caused a 25% decrease in the number of Sholl intersections in vehicle-treated SHAM male mice (*p* = 0.0045 vs. vehicle saline) and a 28% increase in dutasteride-treated SHAM male mice (*p* = 0.0026 vs. DUT saline). DUT left the microglial number of Sholl intersections unchanged in saline treated SHAM male mice. GDX male mice (*n* = 19) also showed significant changes in the number of Sholl intersections (MPTP: F (1, 181) = 25.58, *p* < 0.0001; DUT: F (1, 181) = 1.199, *p* = 0.2751; interaction: (1, 181) = 0.1028 *p* = 0.7489). MPTP caused a 24% decrease in the number of Sholl intersections in vehicle-treated GDX male mice (*p* = 0.0013 vs. vehicle saline) and 25% in dutasteride-treated GDX male mice (*p* = 0.0037 vs. DUT saline). SHAM females (*n* = 18) showed no significant changes in the number of Sholl intersections (MPTP: F (1, 122) = 0.2532, *p* = 0.6157; DUT: F (1, 122) = 4.503, *p* = 0.0359; interaction (1, 122) = 0.09714, *p* = 0.7558) as well as in GDX females (*n* = 15) (MPTP: F (1, 101) = 0.1011, *p* = 0.7511; DUT: F (1, 101) = 7.31, *p* = 0.0080; interaction: F (1, 101) = 0.2882, *p* = 0.5926).

#### 2.4.6. MPTP Caused a Decrease in the Striatal Microglia Radius in Male Mice

Figure 4F shows the maximum reach from the nucleus (radius) of each microglia. Under basal conditions, striatal radii were not different between male and female SHAM and GDX mice (Sex: F (1, 155) = 1.602, *p* = 0.2075; Gonadal status: F (1, 155) = 0.6722, *p* = 0.4136; interaction F (1, 155) = 0.4133, *p* = 0.5212).

In SHAM male mice (*n* = 17), the radius was significantly changed (MPTP: F (1, 173) = 11.77, *p* = 0.0008; DUT: F (1, 173) = 4.373, *p* = 0.0380; interaction F (1, 173) = 0.2283, *p* = 0.6334). MPTP caused a 12% decrease in the radius of vehicle-treated SHAM male mice (*p* = 0.0165 vs. vehicle saline) whereas dutasteride-treated SHAM male mice had no significant decrease (*p* = 0.2117 vs. DUT saline). DUT left the microglial radius unchanged in saline treated SHAM male mice.

GDX male mice (*n* = 19) also showed significant radii changes (MPTP: F (1, 181) = 19.25, *p* < 0.0001; DUT: F (1, 181) = 5.119, *p* = 0.0249; interaction: (1, 181) = 0.1148, *p* = 0.7351). MPTP caused a 14% decrease in the radius of vehicle-treated GDX male mice (*p* = 0.0014 vs. vehicle saline) and 11% in dutasteride-treated GDX male mice (*p* = 0.0480 vs. DUT saline). SHAM females (*n* = 18) did not show significant changes in the radius (MPTP: F (1, 122) = 0.5757, *p* = 0.4495; DUT: F (1, 122) = 2.646, *p* = 0.1064; interaction (1, 122) = 2.071, *p* = 0.1527) as well as GDX females (*n* = 15) (MPTP: F (1, 101) = 1.749, *p* = 0.1894; DUT: F (1, 101) = 1.367, *p* = 0.2451; interaction: F (1, 101) = 0.1519, *p* = 0.6976).

## 3. Discussion

The present study reports a detailed morphological analysis of microglia sex differences in a mouse model of PD using high magnification three-dimensional images to assess in depth microglia characteristics.

**Figure 5:**
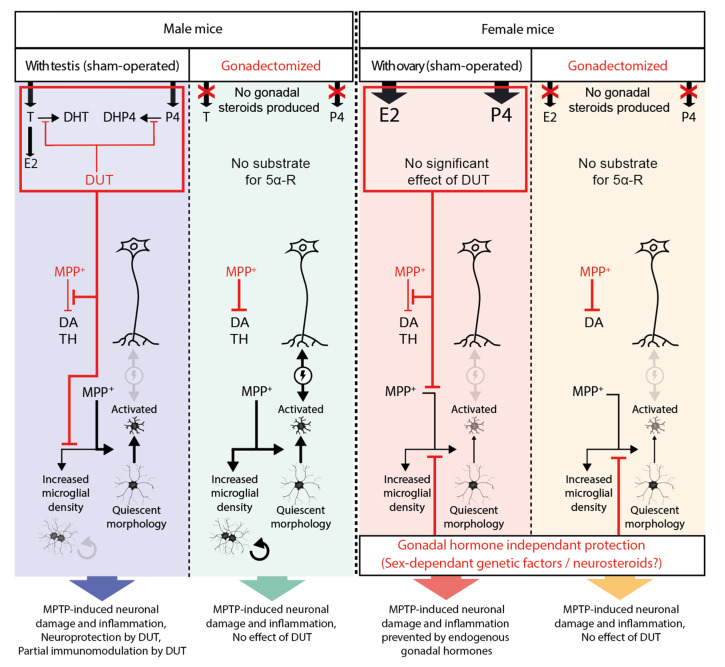
Schematic summary of the results of the present study. Black arrows represent induction, and their thickness shows their significance. Red lines represent inhibition.

Our previous study [55] showed that the MPTP-induced decrease of DA markers and astrogliosis were prevented by sex hormones. In the present study, we showed that both biological sex and sex hormones modulated microgliosis in MPTP mice. In SHAM male mice, we found that DUT partly prevented the MPTP-induced increase in microglial density but did not have an impact on morphological changes from quiescent to activated status. In GDX male mice, MPTP caused microglial activation and increased microglial density. In SHAM and GDX MPTP female mice, striatal microglia measures were at control values suggesting gonadal hormone independent mechanisms at this time after MPTP administration (results summarized in Figure 5). The latter likely implicates genetic sex differences and/or epigenetic mechanisms [66].

Furthermore, we detected and analyzed microglial cells in division resulting in microglial doublets in MPTP-lesioned SHAM and GDX male mice, and this was prevented by DUT treatment in the SHAM male group.

Four animal groups were used (SHAM male, GDX male, SHAM female and GDX female mice) to investigate the effect of biological sex, the effect of sex hormones and lack thereof allowing to model hormonal loss occurring in menopause and andropause. A pre-symptomatic model of PD using a low dose of MPTP was used to get a moderate striatal DA loss and no/or limited changes in DA markers in the substantia nigra and no motor behavior changes. Indeed, we use this pre-symptomatic model to mimic early stages of PD where DA neurons are impaired; this is a good time to intervene to stop and/or delay disease progression. Since female mice are less affected than male mice in our MPTP paradigm and mice motor behavior was unchanged in males as we reported with this paradigm [67], then the same was expected (no change) in female mice.

Inhibiting the 5α-reductase enzyme with DUT can lead to an accumulation of protective female sex hormones in SHAM male mice. Unlike humans, in which steroidogenesis occurs in gonads and adrenal glands, sex steroids in mice are mostly produced in the gonads. Therefore, mouse gonadectomy leads to a major depletion in gonadal steroids, hence 5α-reductase inhibition by DUT has no impact on sex steroid levels in GDX mice. Thus, GDX male and female mice groups allowed evaluating whether DUT could have other activities than modulating sex hormones levels. In the time frame of the MPTP lesion and DUT treatment of our experiment, DUT did not display protective effects in MPTP-lesioned GDX mice, suggesting that the DUT effects were gonadal hormone-dependent.

In SHAM female mice, female sex hormone levels were already high enough to exert protective effects, and DUT treatment would not have any significant impact on the level of protective female sex hormones (Figure 5).

In the present study, MPTP caused a loss of striatal TH^+^ intensity in vehicle-treated SHAM male mice compared to the control group. As we reported previously, DUT exerts a protective effect against MPTP toxicity on DA neurons in non-gonadectomized male mice when administered before the lesion [55,65,67,68]. In GDX males, MPTP caused a decrease of TH intensity, and DUT did not show a protective effect. In SHAM female mice with the protective effect of endogenous female sex hormones, TH intensity was not affected by MPTP. In GDX females, TH intensity tended to be lower in the MPTP-lesioned groups. A previous study in these mice [55] showed higher striatal DA contents in female SHAM and GDX mice than SHAM and GDX male mice but no difference for the DA transporter (DAT) and the vesicular monoamine transporter 2 (VMAT2) specific binding in the striatum and substantia nigra as observed here with TH^+^ intensity.

There is little information on sex-associated morphological and/or functional changes in the microglia of the nigrostriatal pathway. Under basal conditions, no difference between male and female microglia cell density and soma size were reported in the striatum of adult 13-week-old C57BL/6 mice [69]. This is in accordance with the present findings in the striatum of SHAM male and female mice showing no statistical difference in microglia density, arborization terminal points, arborization branch points, arborization segments, arborization length, Sholl intersection points and radii. Moreover, we observed no statistical difference with gonadectomy on these microglia parameters.

MPTP caused an increase in microglial density in male mice only. Moreover, DUT protected against the MPTP-induced increase of striatal microglial density in SHAM male mice. In female mice, there was no increase in microglial density in SHAM and GDX groups, suggesting a gonadal hormone-independent protection.

In male animals, DUT treatment was protective likely because of its modulation of endogenous of gonadal hormones since this protection was observed in SHAM but not in GDX mice. DUT could either act directly on microglia or indirectly by protecting DA neurons and impeding the pro-inflammatory response between neurons and microglia [70]. Alternatively, DUT could modulate microgliosis through a reduction in striatal astrogliosis as previously reported in these SHAM male mice [55].

We previously reported that gonadal hormones prevented the MPTP-induced astrogliosis and DA marker loss in these mice [55]. Interestingly, in the present study, we show that female biological sex displayed a protective effect on striatal MPTP-induced microgliosis (Figure 5). This could be because, unlike other cell types, microglia sex-specific features are determined during brain development and unlikely to change during the life course, even with hormonal variations. This possibility is supported by a study of microglial transplantation in mice of both sexes [61] where MCAO was more severe in male mice and female microglia transplanted to male mice brain still had the ability to protect against MCAO as it did in female mice [61]. A recent detailed study reported that midbrain microglia have a distinct transcriptional profile from microglia in the prefrontal cortex and striatum [71]. Midbrain microglia was associated with a more immune-vigilant transcriptional profile while the prefrontal cortex and striatum microglia profiles were more focused on the expression of genes involved in synaptic remodeling and neuronal architecture [71]. In addition, these authors showed that certain transcripts associated with synaptic transmission (glutamatergic and GABAergic synapses) were higher in females but only in the midbrain [71]. Overall, these results suggest that male microglia show a more inflammatory transcriptional profile in the striatum, midbrain and prefrontal cortex than female microglia [71].

There is clear supporting evidence in rodents of sex differences in the physiology of microglia with respect to the number, morphology and expression of genes; these differences are heterogeneous and more prominent in certain brain regions, such as the hippocampus and amygdala [66,72]. The most striking sex differences were observed more particularly in the early postnatal periods and are less obvious in young adults [72,73]. Studies have shown that some sex-related characteristics of microglia are retained in young adulthood. Indeed, a study showed that juvenile and adult female rats have a greater number of microglia with an amoeboid morphology than male rats in the parietal cortex, hippocampus and amygdala [74]. In addition, sex differences in gene expression for several cytokines as well as chemokines and their respective receptors were observed in the parietal cortex/hippocampus of adult rats and particularly with higher levels of IL-1β in females [74].

With MPTP-induced toxicity and its associated inflammation, sex differences in microglia responses were observed in the present study. Male and female microglia may have distinct mechanisms to respond to MPTP-induced toxicity and associated inflammatory insult. Interestingly in the MCAO mouse model, phagocytic microglia in the female express significantly more phagocytic pathway genes and phagocytic associated functions when compared to males [61]. In MCAO 12-week-old C57BL/6 male mice microglia were reported to express more NF-κB regulated genes and to be more prone to inflammatory activation than females [61]. These differences can also be maintained in vitro and thus do not totally depend on sex steroids. Females can even maintain sex-specific microglial differences when transplanted into the opposite sex brain, and this protects the males from ischemic stroke [61].

Microglia express estrogen receptor beta but few labeling markers for estrogen receptor alpha are present [75]. In the striatum, estrogen receptor alpha and beta and G protein-coupled estrogen receptor 1 (GPER1) are found on glial cells [76]. Glial cells are reported to mediate the neuroprotective actions of gonadal hormones, such as 17β-estradiol and progesterone [77]. Under condition of inflammation or brain injury, steroid (estrogen, progesterone, testosterone) receptors are increased in glial cells; hence these receptors are well positioned to modulate the reactive gliosis and the inflammatory response of glial cells [77,78].

Using a three-dimensional analysis of microglial morphology, we detected and quantified microglial doublets. These doublets occur after the division of microglial cells during their proliferation process. Microglia have a high capacity to autonomously self-renew themselves. Microglial repopulation has been recently investigated in a study characterizing ontogeny and maturation in the adult visual cortex [79]. It was observed that during the repopulation phase following a microglial depletion, microglial cells can occasionally split to two daughter cells and that this process can lead to mature microglia faster than the normal developmental features [79]. Moreover, this study shows that, after their spatial separation, the two daughter cells can be integrated within the microglial network [79].

In the present study, we observed dividing microglial cells when their somas were distinct but still in close contact (Video S2). To our knowledge, this is the first observation of microglial doublets in a MPTP mouse model of PD. Density of striatal microglia doublets showed no statistical difference between SHAM male and female mice. GDX in female mice left unchanged striatal microglia doublet density whereas male mice had a higher density than SHAM male mice. As observed for microglial density, microglial doublets were statistically more abundant in all male mice treated with MPTP compared to saline-treated animals, and DUT prevented this increase in SHAM male mice. This result shows that, when the microglial density increases in response to MPTP toxicity, some of these microglial cells will proliferate. In female mice, the doublet counts were low and remained unchanged by gonadectomy, lesion with MPTP or treatment with DUT. Our results suggest that male gonadal hormones show some protection to reduce microglia proliferation (formation of doublets) whereas in females, both sex and female sex hormones kept microglia doublets low. The MPTP lesion is a more intense insult and increased striatal microglia doublets in both SHAM and GDX male mice whereas SHAM and GDX female mice were protected and showed no increase. Our results suggest that microglia in female mice are less sensitive to MPTP toxicity, which seems to be a biological sex-specific feature. However, in SHAM male mice, DUT modulation of gonadal sex hormones reduced the microglial response to MPTP, which could result from the decrease in astrogliosis and/or DA neuronal damage following DUT treatment.

Microglial cells have a distinctive appearance depending on their activation status. Quiescent microglial cells have small soma and long, highly branched processes. They are highly dynamic and can constantly extend and retract their processes, allowing them to survey their immediate environment [13,80]. Activated microglial cells present an amoeboid shape with a larger soma and some short branches and are able to respond to proinflammatory stimuli [13,80].

Microglial morphology was characterized in the present study using a three-dimensional reconstruction of whole microglial cells from high resolution confocal microscopy imaging. For this purpose, the following six parameters were evaluated: (i) arborization terminal points, (ii) branch points, (iii) segment counts that provide information on the number of microglial ramifications, iv) the arborization length, (v) the number of Sholl intersections and (vi) radius describing the extent of these ramifications. The lower the values of these parameters, the more microglial cells will be of amoeboid shape and will be characteristic of activated microglia. In MPTP-lesioned male mice, microglial cells will present a more activated phenotype with less arborized and shorter ramifications than in non-lesioned male mice. Although DUT was able to prevent microglial proliferation in MPTP-lesioned SHAM male mice, it had no incidence on microglial morphology. Moreover, MPTP had no effect on microglial morphology in female mice. Thus, microglial morphological changes were dependent on the female biological sex but not on gonadal sex hormones (Figure 5). Epigenetic mechanisms including DNA methylation, histone modifications and long non-coding RNAs may also partly explain these sex differences of microglial morphological changes [66]. Of great interest, microglial sex-specific features have already been shown to be defined during brain development with the surge of estradiol in male mice, and microglia have an active contribution in brain masculinization [14,57,58]. Our results suggest that microglial cells in female mice are less sensitive to MPTP toxicity than microglial cells in male mice. Activated microglia and altered neurons can interact negatively with each other to promote neuroinflammation and neurodegeneration [70]. The female sex can have a protective effect against MPTP-induced activated microglial morphology, as shown in Figure 5. Therefore, we can hypothesize that these negative interactions of microglial cells with neurons did not occur in GDX female mice given the quiescent morphology of microglial cells. This could explain the observed beneficial impact of biological sex on TH striatal level in MPTP-lesioned GDX female mice. Taken together, these results suggest that microglia in female mice are less sensitive to MPTP toxicity compared to males, independently of their gonadal status.

Genes localized on the X and Y chromosomes may contribute to gonad-independent, sex-specific neuroprotective effects [77]. Sex chromosome genes could influence the manifestation of PD [81]. The Y chromosome genes sry expressed in the adult brain exerts gonad-independent functions. Sry is aberrantly upregulated in the substantia nigra (SN) of 6-OHDA lesioned male rats in an experimental model of PD. Interestingly, silencing sry in the SN of male rats decreased mitochondrial dysfunction, neuroinflammation, DA cell loss and motor deficits in the 6-OHDA model [81].

We hypothesized that beneficial DUT effects could be mainly mediated by an elevation of endogenous female sex hormones in SHAM male mice. However, we cannot rule out that a decrease of DHT or even of 5α-dihydroprogesterone levels resulting from 5α-reductase inhibition could be beneficial in SHAM male mice. Indeed, some studies report that DHT can be detrimental during the course of PD [82]. However, other studies have shown that DHT supplementation had no effect in different mouse models of PD [82,83].

In GDX mice, DHT and 5α-dihydroprogesterone circulating levels are expected to be depleted because of the absence of compensatory steroidogenesis in the adrenal cortex [84]. The absence of these steroids had no effect on MPTP toxicity since no difference in response to MPTP lesion was observed between SHAM and GDX vehicle-treated male mice. Hence, the decrease of DHT and 5α-dihydroprogesterone levels caused by the pharmacological 5α-reductase inhibition seems not to have a significant impact on MPTP-induced inflammation and neurodegeneration.

In a previous study in the putamen of MPTP monkeys (6 months after MPTP administration), we reported double immunofluorescent staining for Iba1 (marker of myeloid cells including microglia) and TMEM119 (microglia-specific) to determine if the increase in Iba1 density observed in MPTP ovariectomized female monkeys compared to controls could result from an infiltration of circulating monocytes in the striatum [85]. This analysis revealed that the proportion of cells that were only positive for Iba1 increases in the sensorimotor region of the putamen of MPTP versus the control monkeys (7.8% in MPTP versus 2.9% in control animals), suggesting that monocytic infiltration is only partially responsible for the increase in Iba1-positive cell density. Hence, MPTP monkeys showed an increased density of Iba1-positive cells in the putamen that was associated with a small, although significant, increase in peripheral myeloid cells (Iba1-positive/TMEM119-negative). Therefore, the changes in Iba1 observed here in MPTP mice are likely, as shown in ovariectomized female MPTP monkeys, mainly from the microglia of brain origin.

Further studies are required to investigate, apart from microglial morphology reported here, their functions and how they are affected by sex, the hormonal status (gonadectomy to model menopause and andropause), their response to lesion (to model PD and associated inflammation) and treatments (endocrine drugs with anti-inflammatory activity). Moreover, additional studies will be necessary to investigate the mechanisms of action of DUT to support its translation for PD. Our results suggest that DUT can partially prevent the striatal MPTP toxicity in a 10-day paradigm. It would be interesting to assess DUT protective effects in a more progressive model of PD, such as in a transgenic animal model to determine whether DUT could delay the onset and slow the disease progression.

Brain region and sex-specific transcriptional profiles of mouse microglia were recently reported [71]. In the striatum, 454 transcripts were differentially expressed by sex (male > female: 288 transcripts; female > male: 166 transcripts). Transcripts enriched in striatal microglia were associated with pathways related to microtubules. Pathway analysis indicated that transcripts enriched in male microglia in frontal cortex, striatum and midbrain were associated with immune-related pathways and G protein-coupled receptor activity. Transcripts more highly expressed in female microglia in frontal cortex, striatum and midbrain were associated with response to selenium ion and the cytoskeleton. The top pathway associated with transcripts more highly expressed in female microglia these brain regions was response to selenium ion. Gene products of these transcripts represent members of a class of Selenium (Se)-dependent proteins that participate in glucose metabolism and protect cells from oxidative stress by reducing reactive oxygen and nitrogen species. Glutathione peroxidase 1 (GPX1) may play a further role in regulating the inflammatory response. For instance, overexpression of GPX1 results in fewer activated microglia after an ischemic injury [86]. Selenium also directly influences the production of testosterone and sex hormones regulate selenium distribution and metabolism [87].

Barko et al. 2022 used isolated microglia under homeostatic conditions to study their sex differences in a transcriptional profile in various brain regions [71]. Future studies are required to assess whether these transcriptional profiles differ when mice are exposed to toxins to seek if males and females exhibit similar alterations. They found that across brain regions, male microglia exhibit a more inflammatory profile and this is in agreement with a previous study examining the transcriptional profile of microglia from the mouse whole brain [61].

A significant inverse correlation was observed between striatal microglia density and DA content in SHAM and GDX male mice whereas no effect of a lesion on DA and microglia density was observed in SHAM and GDX female mice. A significant inverse correlation was also observed between striatal microglia doublet density and DA content in SHAM and GDX male and female mice. This shows a close link between striatal MPTP-induced DA loss and inflammation as well as the effect of dutasteride or lack thereof.

The present study generated a comprehensive set of data on microglia morphology that showed a gonadal hormone-independent protective phenotype in female mice. We detected and quantified, for the first time in the MPTP mouse model, microglial doublets in a pathological context. We investigated MPTP-induced microglial changes and determined whether microglial proliferation and activation were mediated by gonadal hormone-dependent and/or -independent mechanisms. We report differences in the response to the toxin and to the treatment dependent on biological sex and gonadal status that could give clues in understanding the disease. This is particularly important to be considered since many PD patients are in a menopausal or andropause state.

## 4. Materials and Methods

### 4.1. Animals

**Figure 6:**
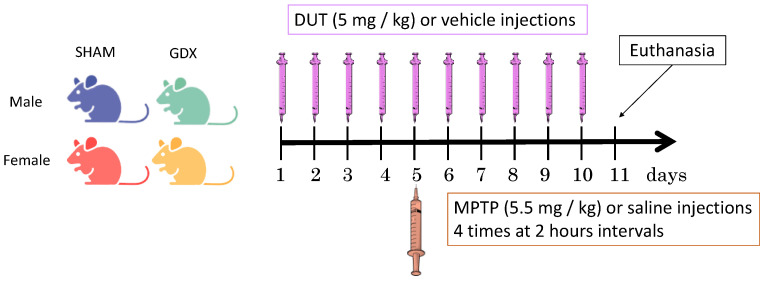
Experimental design. Male and female mice were gonadectomized (GDX) or sham operated (SHAM) and received intraperitoneal injection of dutasteride (DUT) or vehicle and 1-methyl-4-phenyl-1,2,3,6- tetrahydropyridine (MPTP) or saline.

Male (*n* = 68) and female (*n* = 82) C57BL/6N mice (Charles River Canada, Montreal, QC, Canada) of 8 weeks of age were purchased for this experiment. All animals were given food and water ad libitum and housed in cages under controlled conditions at 22 °C with a 12 h light/12 h dark cycle. A sham surgical procedure, the same as for gonadectomized (GDX) mice but without removing the gonads, was performed for sham controls on 34 male and 51 female mice [55]. Animals were allowed to recover for nine weeks before starting the experiments. All efforts were made to minimize animal suffering during the surgery. These mice were then divided into 16 experimental groups including: male (shown in blue) and female (shown in pink) sham-operated mice (SHAM) treated with saline + vehicle (8 males and 10 females), MPTP + saline (9 males and 13 females), saline + dutasteride (9 males and 12 females) and MPTP + dutasteride (8 males and 10 females) and also male (shown in green) and female (shown in yellow) gonadectomized mice (GDX) treated with saline + vehicle (8 males and 8 females), MPTP + saline (9 males and 7 females), saline + dutasteride (8 males and 8 females) and MPTP + dutasteride (9 males and 8 females) (Figure 6). The number of mice per group was based on our previous experiment in the brain of intact male mice that received MPTP and dutasteride treatment [67]. More intact female mice were included in the present experiment to consider that they were at random stages of the estrous cycle, and additional animals were included in the lesioned and treatment groups to consider their possible variable response. The Université Laval Animal Care Committee approved these animal studies (2019-95, CHU 18-039). All efforts were made to minimize animal suffering and to reduce the number of mice used. Effects of the MPTP lesion and dutasteride treatment on DA and astrocyte markers in the brain of these mice were previously reported [55].

### 4.2. Drug Treatment

Mice that received dutasteride (Toronto Research Chemicals, Toronto, ON, Canada) (5 mg/kg, intraperitoneal) or vehicle only (0.9% saline with 1% tween 80, i.p.) were injected once daily for 10 days. On the fifth day of dutasteride/vehicle treatment, mice received four intraperitoneal injections (5.5 mg/kg) at 2-h intervals of acute treatment with MPTP (Sigma Chemical, St. Louis, MO, USA) or saline (Figure 6). The MPTP dose of the present study was chosen to produce a partial lesion of the DA nigrostriatal pathway as reported for these SHAM male mice [55] in order to model early stages of PD in humans. Dutasteride was investigated in the present study at a dose that protected against MPTP toxicity brain DA markers of male mice [67].

### 4.3. Brain Tissue Preparation

On day 11, mice were deeply anesthetized with ketamine-xylazine before cardiac puncture and transcardial perfusion with 0.1 M PBS (137 mM NaCl, 2.7 mM KCl, 4.3 mM Na_2_HPO_4_, 1.47 mM KH_2_PO_4_, pH 7.4). The brain was quickly removed and separated in two hemispheres. The first hemisphere was rapidly placed in isopentane on dry ice (−40 °C) and was used for measures of brain DA markers [55]. The second hemisphere was post-fixed with 4% paraformaldehyde for 48 h, placed in 0.1 M PBS and stored at 4 °C. Fixed brains were frozen with dry-ice/ethanol mixture, mounted on a microtome (Leica Micro-systems Inc., Richmond Hill, ON, Canada) and cut into 25 μm thick coronal sections. The collected brain sections were immersed in tissue cryoprotectant solution (0.05 M PBS pH 7.3, 30% ethylene glycol and 20% glycerol) and stored at −20 °C until use for immunofluorescence.

### 4.4. Tyrosine Hydroxylase (TH) and Iba1 Immunofluorescence

TH intensities and Iba1 positive cell number were measured using an immunofluorescence-based approach. Free-floating 25 μm thick brain sections containing the striatum (4-7 brain sections per mice, 7 to 16 mice for TH immunofluorescence and 4 to 6 mice for Iba1 immunofluorescence) were selected and were first incubated overnight at room temperature with primary antibody sheep anti-TH (Abcam; catalogue no. Ab113; dilution1/200) and rabbit anti-Iba1 (Cederlane; catalogue no. 130300; dilution 1/250). The next day, brain sections were stained with secondary antibody donkey anti-sheep Cy3 (Millipore; catalogue no. AP148C; dilution 1:1000) and donkey anti-rabbit Alexa Fluor 488 (Life technologies; catalogue no. A21202; dilution 1:1000). DAPI nuclear counterstaining (Invitrogen Corporation) was performed to detect nuclei. Slide scanning was performed with an Axio Scan.Z1 Digital Slide Scanner (Zeiss, Toronto, ON, Canada), and images were acquired with a 5X objective lens (NA 0.25). The contour of the striatum was drawn, and TH intensities were assessed by measuring the mean pixel intensity in the delimited areas using MathWorks Matlab^®^ 2018a software. TH measurements in the substantia nigra of these mice were previously reported [55].

For microglial analysis, images were acquired using an Olympus IX81 FV 1000 confocal microscope at a 60× magnification with an additional 1.2 optical zoom. Four image stacks per brain from 3 to 6 mice per experimental group were acquired randomly in a TH positive area indicating the striatal region. Image stacks included 21 z-plans, representing a final volume of 0.0008 mm^3^.

The microglial density was calculated by the counts of all Iba1 positive cells per image stacks.

For the microglial doublet counts, a doublet was defined as two microglial cells with contacting cell bodies in which the physical separation of the nucleus can be observed (Video S2). DAPI staining was used to determine nuclei position and shape within the microglial cell bodies.

### 4.5. Microglia 3D Morphological Analysis

IMARIS^®^ software (Bitplane, Switzerland, version 7.5.1) was used for three-dimension microglial reconstruction from confocal image stacks. For this analysis, only microglial cells with their untruncated cell body within the stack were selected. IMARIS^®^ was used to rebuild microglial cell volumes and then the filament reconstruction tool was used to detect the cell arborization and generate several morphological parameters that can be measured for each microglial cell (Figure S1; Videos S1 and S2).

### 4.6. Statistical Analysis

The statistical analyses used GraphPad Prism software (v.7.0a) for Macintosh Computer. A two-way ANOVA was performed with a Tukey’s post-hoc test. A log transformation of the data was performed in the statistical analyzes of doublet counts (testing the effect of sex and gonadectomy) to homogenize the variance of the groups. A simple regression model was used to determine the coefficient of correlation. A *p* ≤ 0.05 was required for the results to be considered statistically significant.

## Data Availability

The data presented in this study are available upon reasonable request from the corresponding authors.

## Supplementary Materials

The following supporting information can be downloaded at: https://www.mdpi.com/article/10.3390/ph16020152/s1, Figure S1: Method for 3D morphological analysis; Video S1: Video describing the 3D reconstruction of microglia; Video S2: Video showing microglial doublet.
