# Supplementary material for: Three-Dimensional Analysis of Sex- and Gonadal Status- Dependent Microglial Activation in a Mouse Model of Parkinson’s Disease
Source: Pharmaceuticals (Basel). 2023 Jan 20;16(2):152. doi: 10.3390/ph16020152 (PMC9967417; doi:10.3390/ph16020152)
Supplement: Supplementary file 1 [file pharmaceuticals-16-00152-s001.zip › FigureS1.pdf]

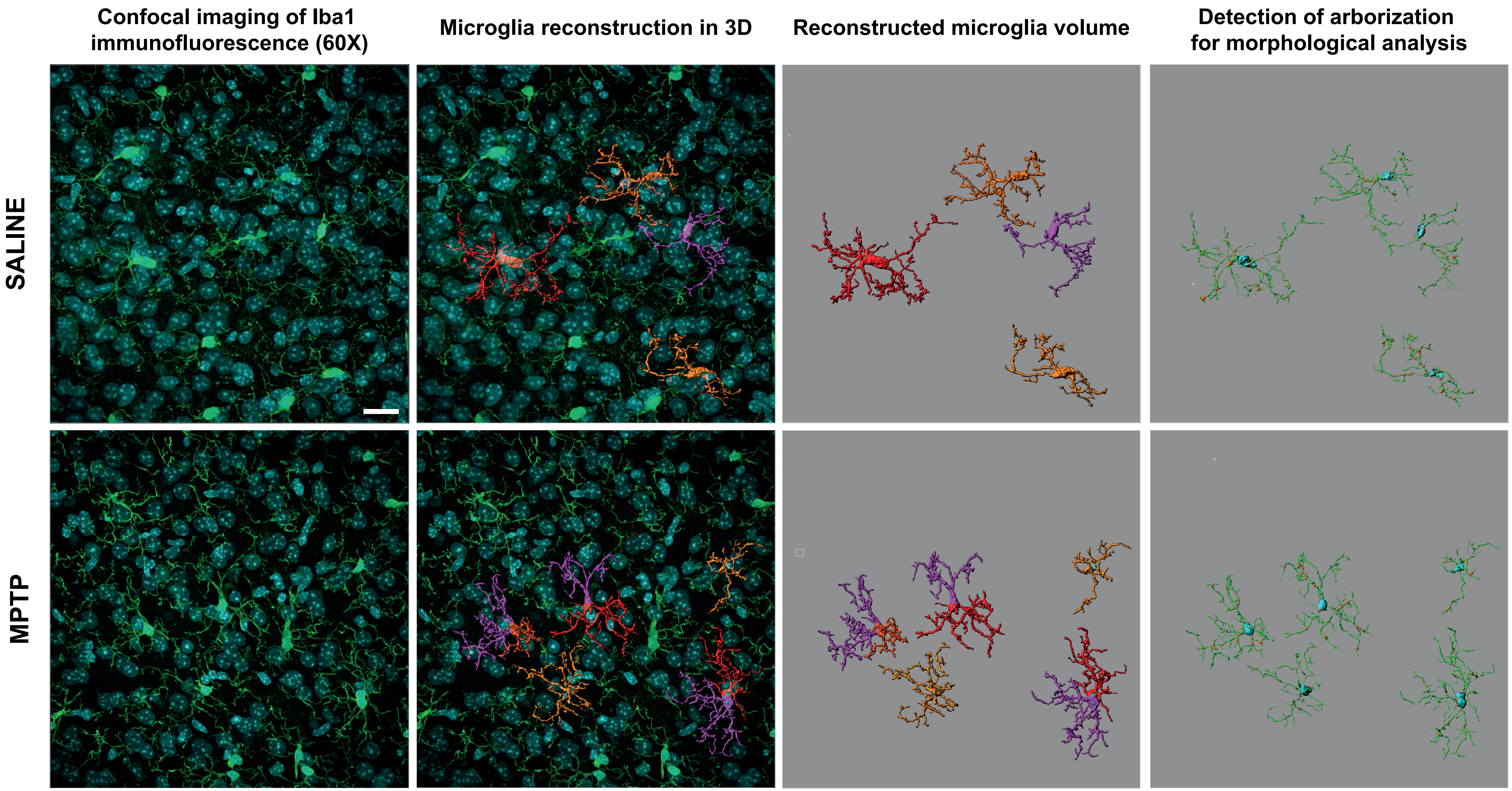

Confocal imaging of Iba1 immunofluorescence (60X)
Microglia reconstruction in 3D
Reconstructed microglia volume
Detection of arborization for morphological analysis
SALINE
MPTP
