# Supplementary material for: Three-Dimensional Analysis of Sex- and Gonadal Status- Dependent Microglial Activation in a Mouse Model of Parkinson’s Disease
Source: Pharmaceuticals (Basel). 2023 Jan 20;16(2):152. doi: 10.3390/ph16020152 (PMC9967417; doi:10.3390/ph16020152)
Supplement: Supplementary file 1 [file pharmaceuticals-16-00152-s001.zip › Suplementary caption.pdf]

Figure S1: Method for 3D morphological analysis. After immunofluorescence using Iba1 staining, the striatal sections were imaged with a confocal microscope at 60X magnification. Images were processed with Imaris® software allowing a reconstruction of the volume of each microglia and their arborization in 3D. Only microglia cells with untruncated cell bodies were analyzed.

Video S1: Video describing the 3D reconstruction of microglia.

Video S2: Video showing a microglial doublet. A microglial doublet was identified as such when the physical separation of the nucleus was seen and when both cell bodies were very close.
